# Microstructure and Mechanical Properties of Mg-Al-La-Mn Composites Reinforced by AlN Particles

**DOI:** 10.3390/ma17143497

**Published:** 2024-07-15

**Authors:** Yuanlin Li, Yuyang Gao, Xiang Zhang, Yan Song, Zhihua Dong, Ang Zhang, Tian Li, Bin Jiang, Fusheng Pan

**Affiliations:** 1 National Engineering Research Center for Magnesium Alloys, College of Materials Science and Engineering, Chongqing University, Chongqing 400044, China; 2 National Key Laboratory of Advanced Casting Technologies, Chongqing University, Chongqing 400044, China; 3 Department of Components and Materials Test & Evaluation Research Center, China Automotive Engineering Research Institute (CAERI), Chongqing 401122, China

**Keywords:** in situ AlN particle, Mg composites, high-temperature mechanical properties

## Abstract

The Mg-Al-RE series heat-resistant magnesium alloys are applied in automotive engine and transmission system components due to their high-temperature performance. However, after serving at a high temperature for a long time, the Al_11_RE_3_ phase coarsened and even decomposed, while the Mg_17_Al_12_ phase grew and dissolved, which limits the service temperature of Mg-Al-RE series heat-resistant magnesium alloys to a maximum of 175 °C. In this study, a new preparation method for in situ AlN particles was presented. The AlN/Mg-4Al-4La-0.3Mn composites were prepared by a master alloy and casting method. The effects of various contents of AlN (0.5–3.0 wt.%) on the microstructure and mechanical properties of the Mg-4Al-4La-0.3Mn (AE44) alloy at room (25 °C) and high temperatures (150–250 °C) were investigated. Microstructure analysis revealed that the inclusion of AlN led to a reduction in both the grain size and second phase size in the AE44 alloy, while also improving the distribution of the second phase. The average grain size, Al_11_La_3_ phase, Al_2_La phase, and Al_3_La phase of the 2.0 wt.% AlN/AE44 composite were 135.7, 9.6, 1.9, and 12.6 μm, respectively, which were significantly lower than those of the AE44 matrix alloy (179.8, 12.6, 3.3, 17.8 μm). The refinement was attributed to the ability of AlN particles to serve as heterogeneous nucleation cores for α-Mg and, at the same time, impede the growth of the solid–liquid interface, eventually leading to grain refinement. With the increase in the AlN content, the mechanical properties of composites initially exhibited an increase at both room and high temperatures, followed by a subsequent decrease. When the AlN content was 2.0 wt.%, the composite exhibited optimal strength and plasticity matching. At room temperature, the TYS, UTS, and EL values of the 2.0 wt.% Mg-4Al-4La-0.3Mn composite were 96 MPa, 175 MPa, and 7.0%, respectively, which were increased by 26 MPa, 27 MPa, and 0.7% when compared with the base alloy. The TYS of the 2.0 wt.% Mg-4Al-4La-0.3Mn composite at 150 °C, 200 °C, and 250 °C were 17 MPa, 14 MPa, and 22 MPa higher than those of the matrix alloy, respectively. The main strengthening mechanisms were second phase strengthening, load transfer strengthening, and thermal mismatch strengthening. At elevated temperatures, AlN particles effectively pinned the grain boundaries, inhibiting their migration, and hindered dislocation climbing, resulting in excellent mechanical properties of the composites at high temperatures. This study contributes to the advancement of in situ AlN particle preparation methods and the exploration of effects of AlN on the properties and microstructure of Mg-Al-RE alloys at high temperatures (150–250 °C).

## 1. Introduction

In response to the need for reduced energy consumption and environmental pollution, there is a growing demand for lightweight materials in industries such as automotives, electronics, and aviation [1]. Magnesium (Mg) alloys with a low density, high specific strength, and excellent damping performance have received significant attention from the industry [2,3,4]. However, Mg alloys still face challenges as a metal structural material, including performance degradation under high-temperature conditions, susceptibility to oxidation, and poor processing performance. To address these shortcomings, scholars often improve the performance of Mg alloys through alloying [5]. The addition of aluminum (Al) can improve the strength of the Mg alloy and enhance its casting performance [6,7,8]. Nevertheless, the Mg_17_Al_12_ phase in Mg-Al alloys exhibits poor thermal stability and is prone to decomposition at high temperatures (above 175 °C) [9], limiting its applications. On the other hand, incorporating rare earth element lanthanum (La) not only reduces casting defects, but also reacts with Al to form Al-La phases with a higher melting point while reducing the formation of the Mg-Al phase [10,11], thereby significantly improving the high-temperature mechanical properties. Consequently, the Mg-Al-La alloy presents promising prospects for applications in high-temperature environments.

Mg matrix composites prepared by incorporating second phase particles are also widely utilized to further enhance the properties of magnesium alloys. In comparison with Mg alloys, Mg matrix composites have superior strength, thermal stability, and corrosion resistance. Ceramic particles serve as one of the most commonly used reinforcing phases in Mg matrix composites, including SiC, TiC, TiB_2_, AlN, and so on [12,13,14,15,16]. Among them, AlN has the closest lattice parameters to the Mg matrix, which can be used as the heterogeneous nucleation core of α-Mg and can play a role in grain refinement [17,18]. Additionally, AlN particles have the advantages of a high melting point (2200 °C), low density (3.26 g/cm^3^), a low thermal expansion coefficient (4.4 × 10^−6^·K^−1^ in the temperature range of 293–673 K), and low thermal conductivity (110–170 W·m^−1^·K^−1^) [19,20]. The incorporation of AlN particles as a reinforcing phase can effectively enhance the elastic modulus and high-temperature mechanical properties of Mg alloys. Chen et al. [21] successfully prepared Mg-Al/AlN composites by adding AlN to the Mg-Al alloy, achieving a tensile strength of 334 MPa and an elongation rate of 4.7% after extrusion. Similarly, Yang et al. [18] prepared in situ AlN particle-reinforced Mg matrix composites by introducing nitrogen into the Mg-Al melt. Compared with the matrix alloy (Mg-9 wt.% Al), the tensile strength and elongation of the as-cast composites increased by 76 MPa and 16.8%, respectively. Therefore, it is of great research value to carry out the preparation of in situ AlN particle-reinforced Mg matrix composites. At present, there is limited research on the preparation of in situ AlN particles and their effects on the room and high-temperature properties and microstructure of Mg-Al-RE alloys.

In this paper, in situ AlN/Mg master alloys are prepared through a Mg_3_N_2_-Al reaction system, and AlN/Mg-4Al-4La-0.3Mn composites are fabricated through a master alloy and casting method. The effects of AlN particles on the microstructure and properties of the Mg-4Al-4La-0.3Mn alloy at room (25 °C) and high temperatures (150, 200, 250 °C) are studied and revealed.

## 2. Materials and Methods

### 2.1. Preparation of AlN/AE44 Composites

**Figure 1:**
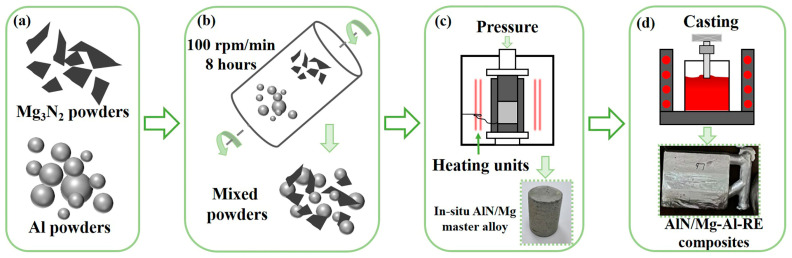
The processing schematic for the AlN/Mg-Al-RE composites: (**a**) Raw powders; (**b**) ball milling; (**c**) hot pressing sintering; (**d**) casting.

Figure 1 represents the processing schematic for the AlN/Mg-Al-RE composites. In this study, a 40 wt.% AlN/Mg master alloy is prepared according to the ${\mathrm{Mg}}_{3}N_{2}+2\mathrm{Al}\to 2\mathrm{AlN}+3\mathrm{Mg}$ reaction system. Al powder (purity > 99.9%, 1~3 μm, Beijing Xingrongyuan Technology Co., Ltd., Beijing, China) and Mg_3_N_2_ powder (purity > 99.9%, 1~2 μm, Shanghai Xiang Tian Nano Co., Ltd., Shanghai, China) are used as raw materials, as observed in Figure 1a. First, 30.44 g of Al powder and 29.56 g of Mg_3_N_2_ powder were uniformly mixed under the protection of argon gas in a planetary ball mill (YXQM-4L, MITR, Changsha, China) at a speed of 100 r/min for 8 h with a ball mill transmission ratio of 0.282, as shown in Figure 1b. The diameter of the 304 stainless steel grinding ball is 6 mm, 8 mm, and 10 mm, and the mass of each diameter is 200 g. The ball powder ratio is 10:1, and the jar capacity is 500 L. Then, the mixed powders are put into the graphite mold for hot pressing sintering at 1000 °C. The pre-pressing operation should be carried out before heating in the sintering furnace. The pre-pressing pressure is 4 t, and the time is 1~2 min. During the sintering process, the heating rate of the instrument is set to 100 °C/min, and the pressure during the heating process is 0.3 t, as shown in Figure 1c.

In this work, the Mg-4Al-4La-0.3Mn (AE44) alloy is selected as the matrix alloy. Furthermore, 0.5 wt.% AlN/Mg-4Al-4La-0.3Mn, 1.0 wt.% AlN/Mg-4Al-4La-0.3Mn, 2.0 wt.% AlN/Mg-4Al-4La-0.3Mn, and 3.0 wt.% AlN/Mg-4Al-4La-0.3Mn composites are successfully prepared by adding commercial Mg (purity > 99.8%), Al (purity > 99.9%), Mg-30 wt.% La, and Mg-5 wt.% Mn master alloys and AlN/Mg master alloys in turn in a resistance melting furnace, as shown in Figure 1d. The melting is carried out under the protection of an SF_6_ and CO_2_ mixed atmosphere, and the melting temperature is 720 °C. After the alloy is completely melted, the AlN/Mg master alloy is added. In order to make the distribution of AlN particles in the melt more uniform, mechanical stirring and ultrasonic treatment are combined for 10 min. Finally, the melt is cast into the metal mold after deslagging, and the ingot of the AlN/AE44 composite is obtained after solidification.

### 2.2. Microstructural Characterization

By using JEOL (Tokyo, Japan) JSM-7800F field emission scanning electron microscopy, the microstructure of the alloy and composite is observed. The acceleration voltage is 15 kV, and the electron beam current is 12 μA. Samples used for SEM observation need to be polished and corroded. The microstructure, size, and composition of the samples are characterized by FEI Talos F200× transmission electron microscopy (TEM) (Thermo Fisher Scientific, Waltham, MA, USA). The Ultima IV X-ray diffractometer (XRD) (Rigaku, Tokyo, Japan) is used to analyze the phase in the sample. The detection angle is 10–90° and the scanning speed is 5°/min.

### 2.3. Mechanical Characterization

**Figure 2:**
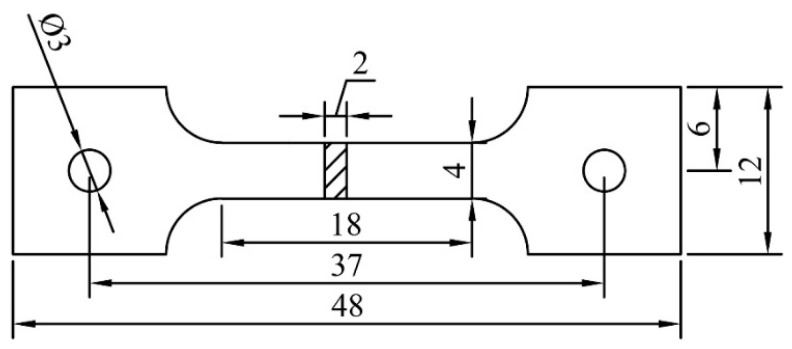
Tensile specimen size at room and high temperatures (mm).

Tensile tests are carried out at room (25 °C) and high temperatures (150 °C, 200 °C, 250 °C). As shown in Figure 2, the plate tensile samples are cut from the as-cast ingots according to the National Standards of People’s Republic of China (GB/T228.1-2010 and GB/T228.2-2015) [22,23]. The gauge length of the tensile sample is 18 mm, and the cross-sectional area is 4 × 2 mm^2^. All tensile samples are tested on the CMT5105-100 KN tensile machine (SUST, Shenzhen, China). The tensile rate is 1.08 mm/min, and each high-temperature tensile sample is held for 10 min. In order to ensure the accuracy of the measurement data, each sample is tested at least 3 times.

## 3. Results and Discussion

### 3.1. In Situ 40 wt.% AlN/Mg Master Alloy

**Figure 3:**
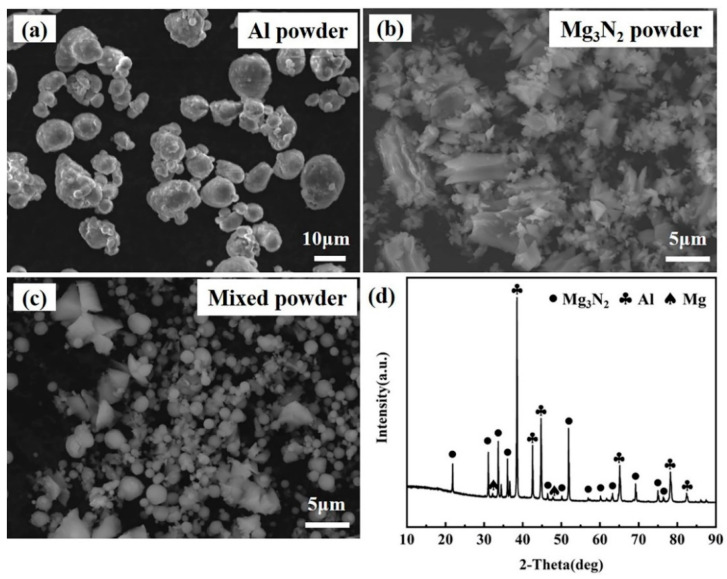
SEM diagrams of raw powders: (**a**) Al powder; (**b**) Mg_3_N_2_ powder; (**c**) mixed powders after ball milling; and (**d**) XRD pattern of (**c**).

The in situ 40 wt.% AlN/Mg master alloy is prepared via the Mg_3_N_2_-Al reaction system. The SEM images in Figure 3a–c depict the morphology before and after powder milling. After ball milling, the mixed Al-Mg_3_N_2_ powders show a significant reduction in particle size (1–10 μm), facilitating the complete reaction during sintering. Additionally, XRD analysis (Figure 3d) indicates that Al and Mg_3_N_2_ phases are predominant phases of the ball-milled mixed powder.

**Figure 4:**
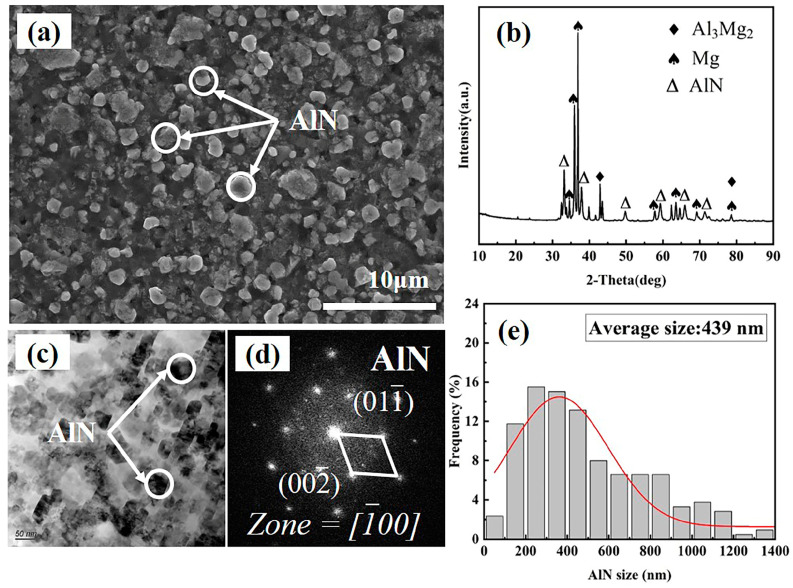
Microstructure of sintered AlN/Mg master alloy: (**a**) SEM image; (**b**) XRD pattern; (**c**) TEM image; (**d**) SAED image; (**e**) statistical diagram of AlN particle size.

The SEM characterization results of the master alloy are presented in Figure 4a. The XRD pattern of the master alloy (Figure 4b) exhibits distinct diffraction peaks corresponding to AlN and α-Mg phases. Furthermore, TEM phase analysis (Figure 4c,d) demonstrates the uniform dispersion of in situ nano-AlN particles within the master alloy without noticeable agglomeration. The statistical analysis of AlN particle size is depicted in Figure 4e, revealing a size range from 40 to 1400 nm, with a predominant distribution between 200 and 600 nm and an average size of 439 nm.

### 3.2. Microstructure of AlN/AE44 Composites

**Figure 5:**
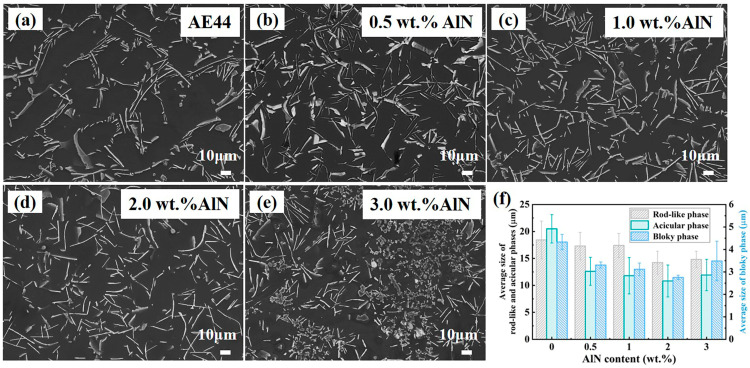
SEM microstructure of AlN/AE44 composites with different AlN contents: (**a**) AE44, (**b**) 0.5 wt.% AlN, (**c**) 1.0 wt.% AlN, (**d**) 2.0 wt.% AlN, (**e**) 3.0 wt.% AlN, and (**f**) second phase size statistics.

**Figure 6:**
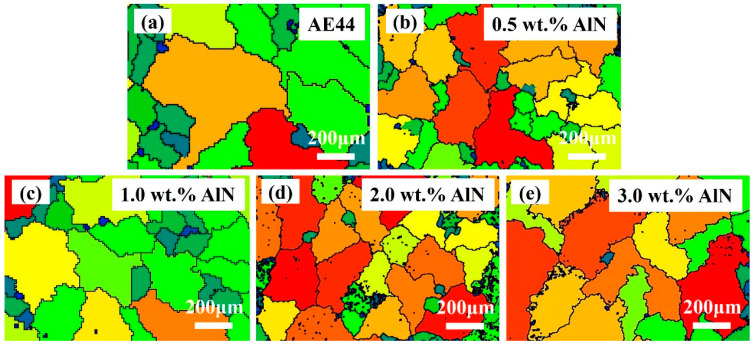
Grain size diagrams of AlN/AE44 composites with different AlN contents: (**a**) AE44, (**b**) 0.5 wt.% AlN, (**c**) 1.0 wt.% AlN, (**d**) 2.0 wt.% AlN, and (**e**) 3.0 wt.% AlN.

AlN particles produced by an in situ reaction are incorporated into the AE44 alloy to fabricate AlN/AE44 composites. The corresponding SEM images and EBSD maps are presented in Figure 5 and Figure 6. As depicted in Figure 5a, the microstructure of the base alloy predominantly consists of the dendritic α-Mg phase and the eutectic phase distributed in a network at the grain boundaries. The morphology of the secondary phases are primarily needle-like and rod-like, with some irregularly blocky shape phases present. Upon the addition of different amounts of AlN, the composite structure is illustrated in Figure 5b–e. Compared to the matrix alloy, the inclusion of AlN improves the distribution of the eutectic phase, leading to a significant reduction in the sizes of both the primary α-Mg phase and secondary phases. As shown in Figure 6, the grain sizes of the AE44 alloy and the 0.5, 1.0, 2.0, and 3.0 wt.% AlN/AE44 composites are 179.8, 169.5, 157.9, 135.7, and 159.5 µm, respectively. The statistical analysis for secondary phase sizes is shown in Figure 5f. The average size for rod-like phases within the matrix alloy and the 0.5, 1.0, 2.0, and 3.0 wt.% AlN/AE44 composites are measured as 17.8 µm, 16.3 µm, 15.9 µm, 12.6 µm, and 13.9 µm, respectively. The average sizes for the acicular phases are measured as 12.6 µm, 11.4 µm, 10.6 µm, 9.6 µm, and 10.5 µm, respectively. The average sizes for the bulk phases are measured as 3.3 µm, 2.4 µm, 2.2 µm, 1.9 µm, and 2.3 µm, respectively. With the increasing AlN content, the size of the grain and secondary phases initially decreases before increasing again. This is attributed to the fact that the in situ generated AlN particles (hcp, a = 0.312 nm and c = 0.4988 nm) possess similar lattice parameters to those of α-Mg (hcp, a = 0.3209 nm, c = 0.5211 nm) [24], which can serve as heterogeneous nucleation cores for the α-Mg grains [18], thereby enhancing the grain nucleation rate. During the solidification process, the hindering effect of nano-AlN particles on the migration of the solid–liquid interface front not only leads to a reduction in the grain size, but also promotes the uniform distribution of solute atoms, thus improving both the distribution and size of the Al-La phase, as depicted in Figure 5 and Figure 6. Zhao et al. [25] found that the AlN particles pinning at the grain boundaries refined the β-Mg_17_Al_12_ phase, significantly improving its distribution. The reduction in the grain and second phase sizes, as well as a more uniform distribution of the second phases, are beneficial for enhancing the mechanical properties of the composite. When reaching an AlN content of 3 wt.%, an obvious accumulation area consisting of granular and multilateral shape phases emerges, with the size of the second phase increasing, as shown in Figure 5e. When the AlN content is at 2.0 wt.%, the refining effect on the secondary phase and grain is optimal.

**Figure 7:**
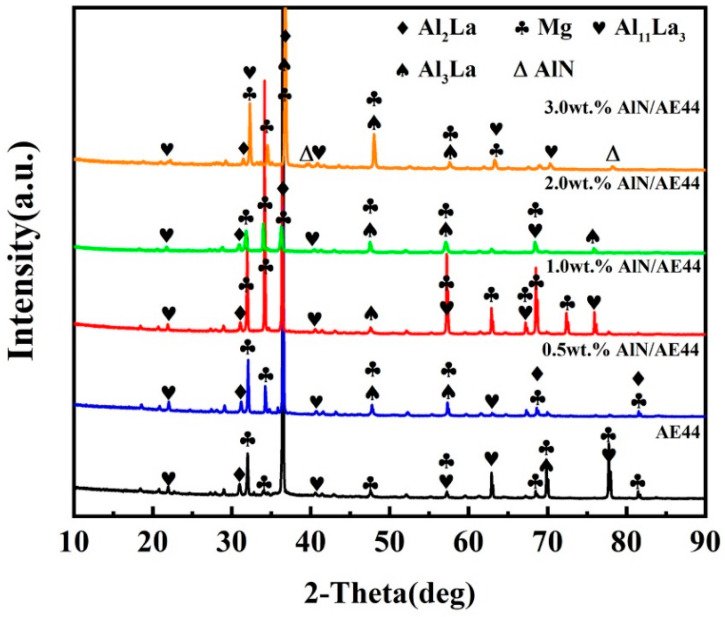
X-ray diffraction patterns of AlN/AE44 composites.

**Figure 8:**
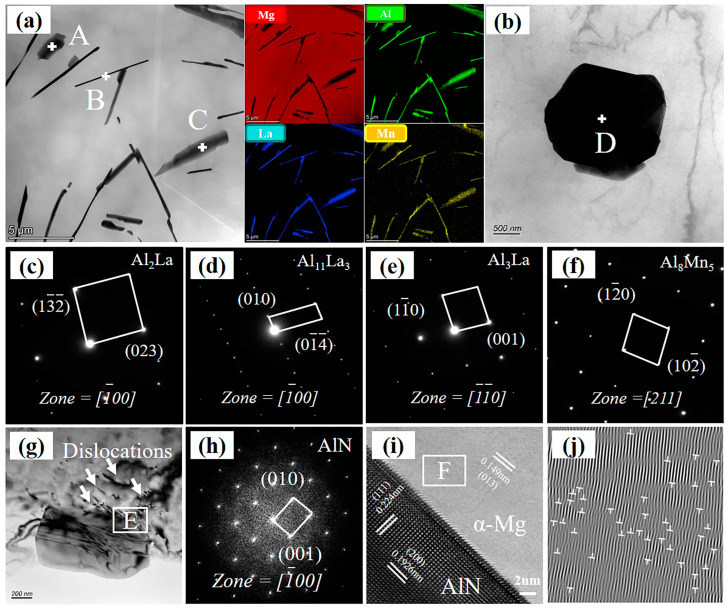
2.0 wt.% AlN/AE44 composite: (**a**) TEM diagram; (**b**) Al_8_Mn_5_ HADDF diagram; (**c**–**f**) SAED diagram of points A–D; (**g**) AlN particles; (**h**) SAED diagram of (**g**); (**i**) high-resolution TEM image of region E; (**j**) inverse fast-Fourier-filtered image of region F.

The XRD pattern of the composites reveals that both the alloy and the composites are predominantly composed of α-Mg, Al_2_La, Al_11_La_3_, and Al_3_La phases, as seen in Figure 7. The TEM morphologies and corresponding SAED patterns of secondary phases in the 2.0 wt.% AlN/AE44 composite are shown in Figure 8. The SAED results (Figure 8c–e) indicate that phases A, B, and C in Figure 8a represent the Al_2_La, Al_11_La_3_, and Al_3_La phases, respectively, with the Al_2_La phase being polygonal, the Al_11_La_3_ phase being needle-like, and the Al_3_La phase being rod-like. Additionally, a small number of independent blocky Al-Mn phases with a size of about 2 μm exist in the composite, as depicted in Figure 8b,f. The result from the SAED pattern (Figure 8f) confirms these to be an Al_8_Mn_5_ phase. Figure 8g shows the dark field transmission image of AlN particles in the composite. It can be revealed that the AlN particles present an irregular multilateral shape with a size of sub-micrometers (~874 nm). The SAED diffraction pattern for the AlN particle is presented in Figure 8h. It was found that there was no obvious interfacial reaction (7i) between AlN particles and the Mg matrix. According to the load transfer mechanism, strong interfacial bonding contributes to improving the tensile strength. Figure 8j shows an inverse fast-Fourier-filtered image of region F. A large number of dislocations are found around the AlN particles, indicating that thermal residual stresses generate during solidification due to differences in the thermal expansion coefficients between reinforcement (4–5 × 10^−6^/K [26]) and the matrix (24.2 × 10^−6^/K [27]), leading to lattice distortion near the AlN/AE44 interface. The increased dislocation density at the particle/matrix interface helps to increase the strength [28].

**Figure 9:**
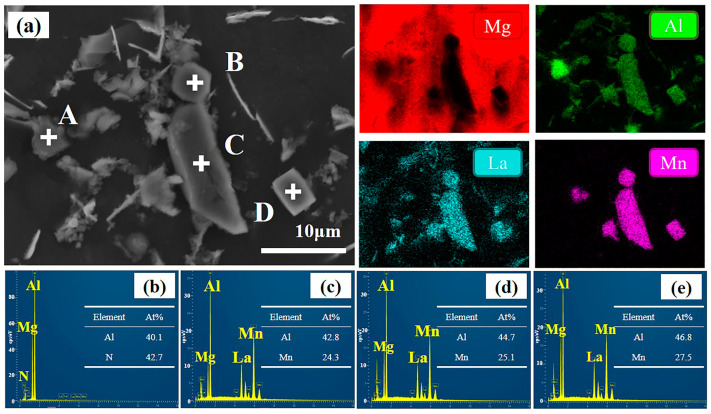
(**a**) SEM image and EDS mapping results of second phases in 3.0 wt.% AlN/AE44 composite; (**b**–**e**) EDS point analysis of A—AlN, B—Al_8_Mn_5_, C—Al_8_Mn_5_, D—Al_8_Mn_5_.

**Figure 10:**
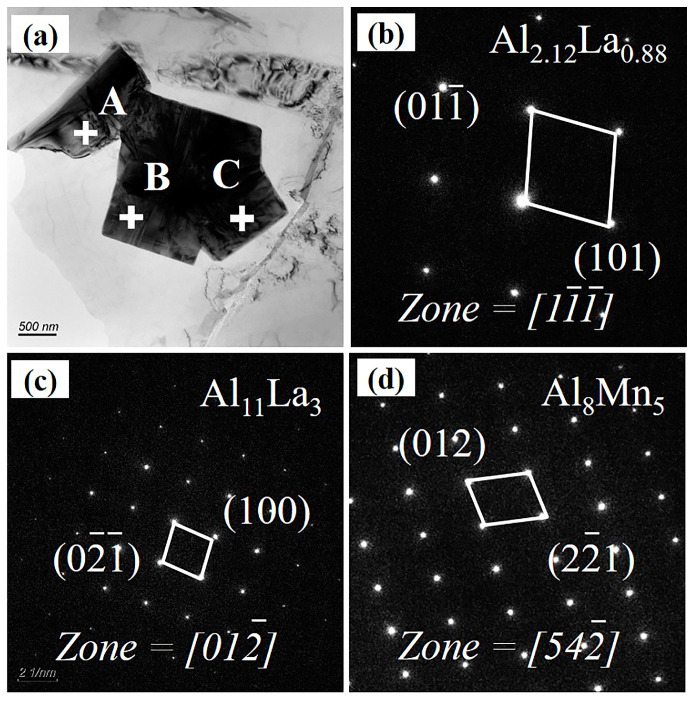
(**a**) TEM image of 3.0 wt.% AlN/AE44, and SAED patterns of (**b**) point A, (**c**) point B, (**d**) point C.

Based on the SEM images (Figure 5), it is evident that the 3.0 wt.% AlN/AE44 composite contains numerous particle-rich regions. The EDS results of the 3.0 wt.% AlN/AE44 composite are presented in Figure 9. The EDS surface scanning and point scanning results confirm that the block phase is Al_8_Mn_5_ phase, with an interlaced Al-La phase in its vicinity. As the AlN content increases from 2.0 wt.% to 3.0 wt.%, the quantity of the Al_8_Mn_5_ phase formed as a result of the reaction between residual Al in the master alloy and Mn in the magnesium melt continues to increase, leading to an enlargement in size from approximately 2 μm to 10 μm. The rich region formed by the coexistence of a large Al_8_Mn_5_ phase and Al-La phase causes stress concentration, leading to more micro-cracks near the agglomeration zone, which has a deteriorating influence on mechanical properties. TEM analysis reveals aggregates composed of Al_2.12_La_0.88_, Al_11_La_3_, and Al_8_Mn_5_ phases, as shown in Figure 10. It is observed that the Al_8_Mn_5_ phase is embedded within the Al_11_La_3_ phase and attached to its growth, indicating a certain orientation relationship between these two phases. Similarly, Qin et al. [29] found that the Al_6_Mn phase had an orientation relationship with the Al_3_La phase ((111) Al_6_Mn//(100) Al_3_La and [110] Al_6_Mn//[010] Al_3_La) in the die-cast Mg-4Al-3La-1Ca-0.3Mn alloy.

### 3.3. Mechanical Properties of AlN/AE44 Composites

#### 3.3.1. Mechanical Properties of AlN/AE44 Composites at Room Temperature

**Figure 11:**
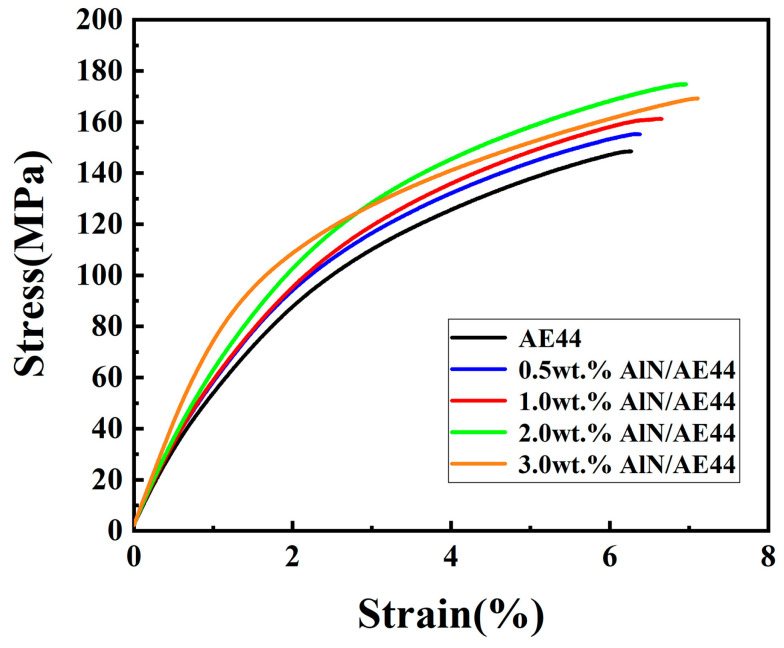
Tensile stress–strain curves of AlN/AE44 composites with different AlN contents at room temperature.

The tensile curves of the AE44 alloy and composites at room temperature are presented in Figure 11, with the statistical properties shown in Table 1. The addition of AlN particles leads to an increase in the strength and plasticity of the AE44 alloy. Specifically, the yield strength (σ_0.2_), ultimate tensile strength (σ_UTS_), and fracture strain (ε) gradually increase as the amount of AlN increases from 0.5 wt.% to 2 wt.%. The maximum values for yield strength (96 MPa), ultimate tensile strength (175 MPa), and fracture strain (7.0%) are achieved by the 2.0 wt.% AlN/AE44 composite. Compared to the matrix alloy, this composite exhibits an increase of 41.4% in ultimate tensile strength and 18.2% in yield strength. Sankaranarayanan et al. [30] have posited that the mechanical strength of alloys is significantly influenced by the density of dislocations, as well as the presence of obstacles that impede dislocation motion. In this study, the second-phase strengthening effect of nano-sized AlN particles is identified as the primary strengthening mechanism, which enhances tensile strength by hindering dislocation movements [31]. In addition, the load transfer strengthening and thermal mismatch strengthening mentioned earlier also contribute to the mechanical properties. However, when AlN particles are added at a higher concentration of 3.0 wt.%, there is a slight decrease in the ultimate tensile strength. This can be mainly attributed to two factors as follows: firstly, the agglomeration of AlN (Figure 9a) weakened the strengthening effect of the reinforced particles; secondly, the presence of a large-sized Al_8_Mn_5_ phase (10 μm) agglomeration zone in the 3.0 wt.% AlN/AE44 composite deteriorated its properties. In contrast, a smaller-sized Al_8_Mn_5_ phase (2 μm) is observed in the 2.0 wt.% AlN/AE44 composite. According to the study of Trang et al. [32], a finer Al_8_Mn_5_ phase (5 μm) could effectively improve alloy strength more than larger sizes (8–10 μm). Therefore, with an increase in AlN addition to 3.0 wt.%, tensile performance begin to decline. Furthermore, SEM images (Figure 5) reveal that enrichment zones formed by the coexistence of the Al_8_Mn_5_ phase and Al-La phase at 3.0 wt.% cause stress concentration and lead to more micro-cracks near the agglomeration zone, further deteriorating the mechanical properties.

**Figure 12:**
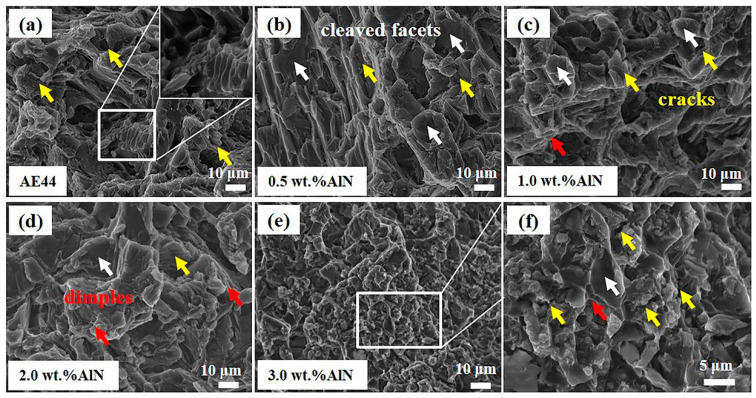
Tensile fracture morphologies of AlN/AE44 composites with different AlN contents at room temperature. (**a**) AE44; (**b**) 0.5 wt.% AlN/AE44; (**c**) 1.0 wt.% AlN/AE44; (**d**) 2.0 wt.% AlN/AE44; (**e**) 3.0 wt.% AlN/AE44; (**f**) partial magnification of (**e**).

The room temperature fracture morphology of AlN/AE44 composites with different AlN contents is shown in Figure 12. The fracture of the AE44 alloy exhibits extensive tearing edges and cleavage planes, indicating that the fracture mode of the alloy is predominantly brittle fracture. With the increase in the AlN content in the composites, the size of the cleavage plane decreases gradually. The presence of dimples is found in the fracture of the 2.0 wt.% AlN/AE44 composite, indicating a mixed fracture mode combining brittle and plastic fractures. The addition of AlN particles contributes to improving the plasticity of the alloy. In the 3.0 wt.% AlN/AE44 composite, stress concentration is prone to occur in the agglomerated region of the Al_8_Mn_5_ phase and Al-La phase, leading to micro-crack initiation, as shown in Figure 12f.

#### 3.3.2. Mechanical Properties of AlN/AE44 Composites at High Temperatures

**Figure 13:**
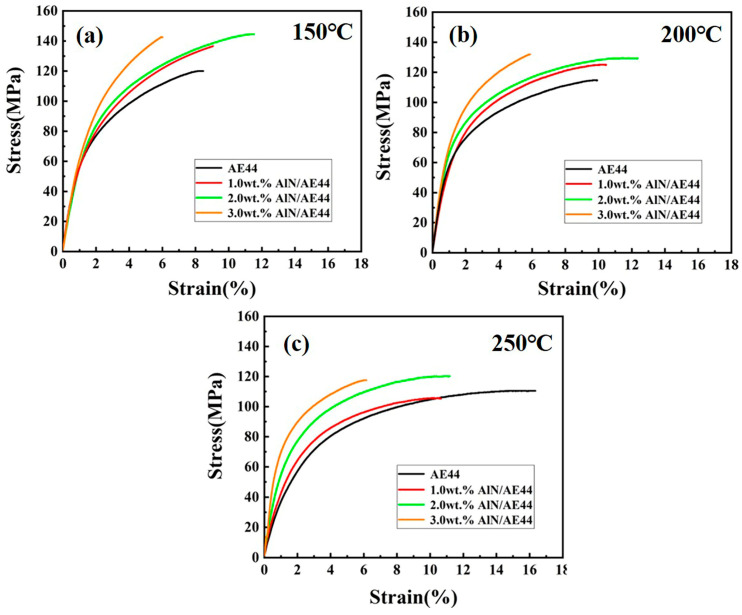
Tensile stress–strain curves of AlN/AE44 composites with different AlN contents at high temperatures. (**a**) 150 °C; (**b**) 200 °C; (**c**) 250 °C.

Figure 13 shows the elevated temperature mechanical properties of AlN/AE44 composites with different contents of AlN particles at 150–250 °C. The statistical results are shown in Table 2. With the increase in the tensile temperature, the softening and slipping of the grain boundaries weaken its blocking effect on dislocation [33], leading to a decrease in the yield strength and ultimate tensile strength of the AE44 alloy and a gradual improvement in fracture strain. With the increase in the AlN particles content, the comprehensive mechanical properties of the composites at 150–250 °C show a trend of increasing first and then decreasing. The 2.0 wt.% AlN/AE44 composite has the best comprehensive mechanical properties. The yield strength, ultimate tensile strength, and fracture strain at 150 °C are 73 MPa, 145 MPa, and 11.5%, respectively, which are 17 MPa, 25 MPa, and 3% higher than those of the matrix alloy (56 MPa, 120 MPa, and 8.5%). At 250 °C, the yield strength (67 MPa) and tensile strength (120 MPa) of the 2.0 wt.% AlN/AE44 composite are 22 MPa and 10 MPa higher than those of the matrix alloy (45 MPa and 110 MPa), respectively. This is due to the fact that nanoparticles with good thermal stability are beneficial for pinning grain boundaries at high temperature and hindering the movement of grain boundaries, which is conductive to improve the high-temperature mechanical properties of the composites [34,35]. Moreover, at high temperatures, dislocations primarily bypass reinforcing particles through climbing. Thus, adding nano-sized AlN particles to the AE44 alloy can impede dislocation movements by increasing climbing stress. This requires more energy for dislocations to complete their climbing process and enhances the tensile strength of the composites. In summary, incorporating nano-sized AlN particles can effectively improve the high-temperature mechanical properties of AE44 composites.

**Figure 14:**
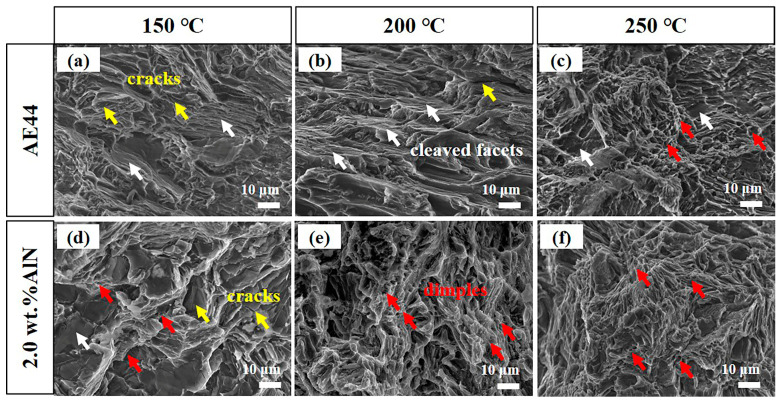
Tensile fracture morphologies of (**a**–**c**) AE44 alloy and (**d**–**f**) 2.0 wt.% AlN/AE44 composite at high temperatures (150, 200, 250 °C).

The tensile fracture morphology of the AE44 alloy at different temperatures (150, 200, 250 °C) is shown in Figure 14a–c. At relatively low tensile temperatures (150 °C, 200 °C), the fracture of the AE44 alloy exhibits typical brittle characteristics, with a large and smooth cleavage plane and a stepped pattern formed by multiple cleavage planes. Upon reaching 250 °C (Figure 14c), distinct dimples and cleavage planes can be observed on the fracture surface of the AE44 alloy, indicating a transition from a brittle to mixed brittle–ductile fracture mode as the temperature increases. Consequently, the high-temperature plasticity of the AE44 alloy improves with the rising temperature. As shown in Figure 13 and Table 2, the fracture strain of the 2.0 wt.% AlN/AE44 composite exhibits little change with the increase in tensile temperature, which is consistent with the fracture morphology of the 2.0 wt.% AlN/AE44 composite at different tensile temperatures (as shown in Figure 14d–f). At a tensile temperature of 150 °C, tear ridges and some ductile holes are visible on the fractured surface of the 2.0 wt.% AlN/AE44 composite, while the AE44 alloy displays larger and more prominent cleavage planes. This indicates superior plasticity for the composite when compared to the matrix.

**Figure 15:**
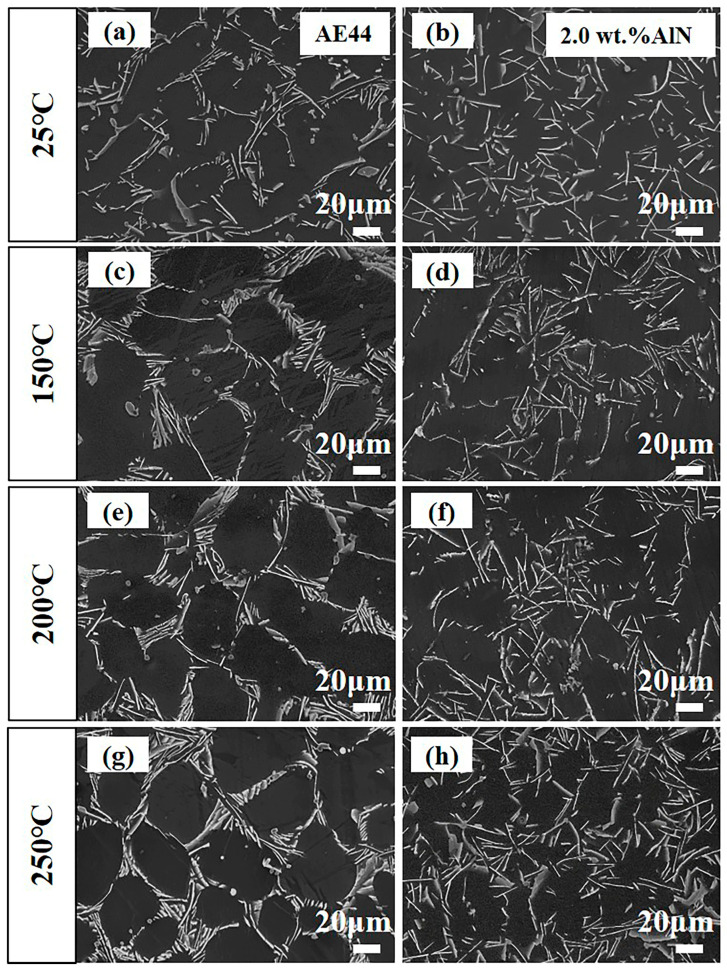
Comparison of the microstructure between (**a**,**c**,**e**,**g**) AE44 alloy and (**b**,**d**,**f**,**h**) 2.0 wt.% AlN/AE44 composite after deformation at different temperatures (25, 150, 200, 250 °C).

Figure 15 illustrates the microstructure of the AE44 and 2.0 wt.% AlN/AE44 composite after undergoing deformation at temperatures ranging from 25 to 250 °C. Following deformation at room temperature, the secondary phase in the AE44 alloy is predominantly distributed in a network and concentrated at grain boundaries, as depicted in Figure 15a. As shown in Figure 15c,e,g, some coarsening occurs in the acicular Al_11_La_3_ phase and the short rod-like Al_3_La phase with increasing temperature, while the quantity of the granular Al_2_La phase gradually decreases and dissolves, which weakened the strengthening effect at high temperatures. It can be observed in Figure 15d,f,h that the secondary phases of the 2.0 wt.% AlN/AE44 composite also undergo growth to a certain extent following deformation at various high temperatures. Compared to AE44, at equivalent temperatures, the distribution of secondary phases within the 2.0 wt.% AlN/AE44 composite is more uniform and smaller, effectively impeding dislocation movement and enhancing high-temperature mechanical properties. The synergistic combination of AlN particles, finer α-Mg grains, and refined Al-RE phases enhanced the tensile properties of the composites at room and high temperatures. At room temperature, the comprehensive tensile properties of the 2.0 wt.% AlN/AE44 composite (TYS: 96 MPa, UTS: 175 MPa, and EL: 7.0%) prepared by the master alloy and casting method were higher than those of the AE44 alloy prepared by Liu et al. [11] (TYS: 79 MPa, UTS: 166 MPa, and EL: 4.0%). Zhang et al. [36,37,38] investigated the high-temperature (200–250 °C) mechanical properties of die-cast Mg-4A1-*x*La-(0.3,0.4)Mn alloys. The results indicated that the UTS of the die-cast alloy ranges from 113 to 126 MPa at 200 °C, while ranging from 92 to 104 MPa at 250 °C. In this study, the UTS of the 2.0 wt.% AlN/AE44 composite is measured at 129 MPa in 200 °C and at 120 MPa in 250 °C, demonstrating excellent mechanical properties under high-temperature conditions.

## 4. Conclusions

1. The addition of AlN particles effectively refines the grain size of the AE44 matrix alloy and reduces the size of the second phases (Al_11_La_3_, Al_2_La, Al_3_La), as well as improving the distribution of the second phase. The average grain size, Al_11_La_3_ phase, Al_2_La phase, and Al_3_La phase of the 2.0 wt.% AlN/AE44 composite are 135.7, 9.6, 1.9, and 12.6 μm, respectively, which are significantly lower than those of the AE44 matrix alloy (179.8, 12.6, 3.3, 17.8 μm).
2. The 2.0 wt.% AlN/AE44 composite shows the optimal combination of strength and plasticity at room temperature, with TYS, UTS, and EL values of 96 MPa, 175 MPa, and 7.0%, respectively. The outstanding mechanical properties of the 2.0 wt.% AlN/AE44 composite can be attributed to the synergistic effects of second-phase strengthening, load transfer strengthening, and thermal mismatch strengthening.
3. At elevated temperatures (150–250 °C), the performance of the composite exhibits minimal degradation with increasing temperature due to the pinning effect of AlN particles on grain boundaries and enhanced dislocation climbing stress, resulting in excellent mechanical properties at high temperatures.

## Figures and Tables

**Table 1:** Tensile properties of AlN/AE44 composites at room temperature.

Samples	σ_0.2_/MPa	σ_UTS_/MPa	ε/%
AE44	70 ± 2	148 ± 3	6.3 ± 0.3
0.5 wt.% AlN	82 ± 1	155 ± 2	6.4 ± 0.5
1.0 wt.% AlN	90 ± 3	161 ± 1	6.7 ± 0.2
2.0 wt.% AlN	96 ± 2	175 ± 2	7.0 ± 0.2
3.0 wt.% AlN	99 ± 4	169 ± 5	7.0 ± 0.4

**Table 2:** Tensile properties of AlN/AE44 composites at room temperature (150, 200, 250 °C).

Samples	150 °C			200 °C			250 °C		
	σ_0.2_/MPa	σ_UTS_/MPa	ε/%	σ_0.2_/MPa	σ_UTS_/MPa	ε/%	σ_0.2_/MPa	σ_UTS_/MPa	ε/%
AE44	56 ± 3	120 ± 4	8.5 ± 0.4	57 ± 4	115 ± 3	10.0 ± 0.3	45 ± 4	110 ± 3	16.3 ± 0.3
1.0 wt.% AlN	63 ± 2	137 ± 3	9.1 ± 0.3	65 ± 3	125 ± 3	10.5 ± 0.4	56 ± 3	105 ± 2	10.7 ± 0.4
2.0 wt.% AlN	73 ± 2	145 ± 2	11.5 ± 0.2	71 ± 2	129 ± 1	12.4 ± 0.2	67 ± 3	120 ± 2	11.2 ± 0.3
3.0 wt.% AlN	79 ± 3	143 ± 4	6.0 ± 0.3	77 ± 3	132 ± 2	5.9 ± 0.4	75 ± 5	117 ± 3	6.1 ± 0.5

## Data Availability

The original contributions presented in the study are included in the article, further inquiries can be directed to the corresponding author.
